# Hard Palate Graft Combined with Fricke Flap: Satisfactory Option for Reconstruction of Extensive Lower Eyelid Defects—A Case Series

**DOI:** 10.3390/jcm14072503

**Published:** 2025-04-07

**Authors:** Paola Parisi, Flavio Andrea Govoni, Tiziano Pallara, Antonio Bonadies, Marinella Tedesco, Elena Rita Govoni, Emilia Migliano

**Affiliations:** 1 Department of Plastic and Regenerative Surgery, San Gallicano Dermatological Institute IRCCS, 00144 Rome, Italy; paola.parisi@ifo.it (P.P.); antonio.bonadies@ifo.it (A.B.); marinella.tedesco@ifo.it (M.T.); emilia.migliano@ifo.it (E.M.); 2 Department of Maxillo-Facial Surgery, San Camillo Forlanini Hospital, 00152 Rome, Italy; flaviogovoni@gmail.com; 3 Campus Bio-Medico, Faculty of Medicine, University of Rome, 00128 Rome, Italy; elenarita.govoni@alcampus.it

**Keywords:** extensive lower eyelid defect, eyelid reconstruction, hard palate graft, Fricke flap, skin cancer

## Abstract

**Background:** The reconstruction of extensive full-thickness lower eyelid defects constitutes a challenge for plastic surgeons. Various techniques have been described to cater to patients’ specific defect needs, with the aim of achieving the best results. **Materials and Methods:** We performed a retrospective observational study assessing our experience with a combination of a single-stage procedure consisting of a hard palate graft and a Fricke flap for patients with complex lower lid resections undergoing immediate total reconstruction at our institution. Clinical data, histological type and results, size of tumor, recurrences, and post-operative complications were collected to evaluate outcomes. A Visual Analogue 10-point scale was administered to all patients to assess esthetic and functional outcomes. **Results:** Seven lower lid reconstructions were performed, with all patients receiving immediate reconstruction. The age of the patients ranged from 55 to 82. Five skin cancers were located on the right side and three on the left side. In all cases, histological diagnosis was non-melanoma skin cancers. The mean size of the tumor was 1.7 × 1.7. In all patients, negative surgical margins were obtained. All patients underwent 24-month follow up. No immediate complication from surgery was recorded within the first 30 days. During follow-up, lower lid ectropion was observed in one patient due to the development of a retracting scar. No local cancer recurrence or nodal metastasis were detected until 2 years follow-up. In only one case, adjuvant therapy was required. The aesthetic results were deemed satisfactory by all patients. **Conclusions:** According to our experience, the combination of a Fricke flap and hard palate graft is an excellent option for total lower eyelid reconstruction, with low morbidity and favorable outcomes, even in elderly and frail patients where satisfactory results were achieved in a single-stage procedure and short operative times.

## 1. Introduction

Eyelid malignancies represent between 5% and 10% of all skin cancers [1]. The most common eyelid malignancies are basal cell carcinoma (BCC), squamous cell carcinoma (SCC), sebaceous cell carcinoma (Seb Ca), Merkel cell carcinoma, and melanoma.

Cook et al. [1] evidenced that the incidence of eyelid tumors is predominant in the white population and determined that BCC is the most common malignant eyelid tumor in white individuals. This finding has been replicated consistently throughout the literature, with BCC representing 85–95% of all eyelid malignancies, SCC representing 3.4–12.6%, Seb Ca representing 0.6–10.2%, and both melanoma and Merkel cell carcinoma representing less than 1.1–8%.

The main risk factor in periocular skin cancers is ultraviolet radiation (UVR) exposure. Ultraviolet radiation induces the formation of carcinogenic photoproducts such as the cyclobutene pyrimidine dimer (CPD) and 6-4 pyrimidine-primidone, which induce DNA mutations [2]. Ultraviolet radiation also causes local immune suppression, which, coupled with DNA abnormalities in both tumor suppressor genes and oncogenes, leads to the development of skin cancers [2].

The standard treatment for all eyelid carcinomas is surgical excision with negative margins, although controversy still exists regarding the recommended margins for each specific malignancy [2]. Given the constraints of the anatomy and function of the eyelids, excision with negative margins and reconstruction can be challenging. In cases of significant tissue invasion or metastasis, complete tumor removal may not be possible.

Preserving the functional integrity of the lower eyelid when facing full-thickness defects is challenging, as this anatomic region is composed of a bilamellar structure: the anterior layer consisting of skin and orbicularis oculi muscle, and the posterior layer formed by tarsus and conjunctiva.

Customizing reconstruction is necessary to provide each patient with the most appropriate surgical approach. Factors such as eyelid laxity significantly influence the choice of procedures and their potential benefits. The method of lower lid reconstruction is determined by the location of the excised lesion, amount of the tissue defect, and age of the patients, and the resection of malignant lesions is one of the most frequent causes of lower eyelid defects, followed by trauma. Considering the normal structure of the lower eyelid, the reconstruction of anterior and posterior lamella by harvesting tissue from various sites has been established.

Repair by direct closure is always favored whenever possible after trauma or malignancy entailing a resection of 25–40% of the lid size relative to the patient’s age [3,4]. However, larger defects require reconstruction with flaps and/or grafts. In the literature, various techniques have been described for the reconstruction of both the anterior and posterior lamella of the lower eyelid, constantly evolving with modifications to achieve the best results according to the characteristics of the defect and patient needs [3]. Proper technique selection is generally based on two factors: size of the defect and involvement of the anterior with or without the posterior lamella [5]. Concerning the posterior lamella, many reconstructive options—both autologous and alloplastic with synthetic meshes—have been used and described [6,7,8].

Autologous hard palate grafts are often considered as a good option because of their ability to provide structural support to the lids; they have an excellent consistency, are firm but still very flexible, and may be obtained in a large size to reconstruct the entire eyelid [6]. Furthermore, an autologous hard palate mucosal graft is very similar to the lower lid tarsus in terms of contour, stiffness, and thickness, with a low risk of rejection [7]. In addition, it seems to be stronger than lip or buccal mucosal graft, precluding parotid duct damage and visible oral scarring [8,9]. Regarding the anterior lamella, different flaps find their use in reconstructive planning, such as an advancement-rotation flap from the cheek, glabella, or upper lid.

The Fricke flap, first described by Jochim Fricke in 1829 [10,11], is a temporally based monopedicle forehead transposition flap. In its original version, the donor site is situated immediately above the eyebrow, with the maximal medial extent delineated by the supraorbital neurovascular bundle. The skin flap is raised and transposed to cover the defect and the donor area can be primarily closed or closed by a skin graft [12]. Even though the Fricke flap is often considered as a last resort option for general lower lid defects, it should be considered as one of the best procedures for wide but short defects, i.e., involving the entire length of the lower lid, but with a short vertical size [12]. The aim of the present article is to describe our experience in complex lower eyelid reconstructions involving anterior and posterior lamellae after the surgical excision of tumors using a Fricke flap in association with a hard palate graft.

## 2. Materials and Methods

A retrospective observational study evaluating all patients undergoing complex total lower lid immediate reconstruction with a Fricke flap and hard palate graft after tumor resection was performed from January 2018 to January 2021 at the Plastic Surgery division—San Gallicano IRCCS, Rome. The study has received Institutional Ethical Committee approval (number 102/ISG/24).

The patient inclusion criteria for the study were as follows: presence of a malignant skin lesion, patient age between 18 and 90, adherence to follow-up at 1, 3, 6, 12, and 24 months follow-up. Exclusion criteria were as follows: previous radiotherapy of the head and neck, benign histological type, patients lost to follow-up before 24 months.

Signed informed consent was obtained from all patients enrolled in this study.

Demographic data, tumor characteristics including findings from histological reports and tumor size, clinical outcomes (i.e., post-operative complications), and oncological outcomes (i.e., recurrences) were collected. A 10-point Visual Analogue Scale (VAS) was administered at 12 months of follow-up, and its results were evaluated (0 = worst esthetic result, 10 = best esthetic result).

Accurate preoperative planning was performed for all patients, including magnetic resonance imaging (MRI) for cases clinically determined to show important adhesion to deep tissues, to rule out orbital invasion.

**Figure 1:**
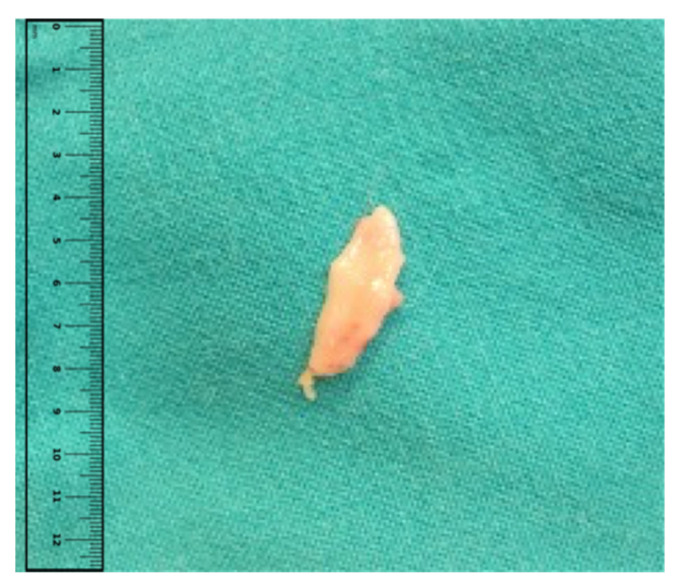
Sample of a hard palate graft for a lower eyelid reconstruction. Usually, a sample of 20 × 8 mm is harvested on the lower eyelid.

Skin marking of the excision area was performed by considering adequate margins for tumor resection in accordance with EDF-EADO-EORTC, AJCC, and NCC guidelines. Additional markings were performed to account for the Fricke flap as well as the hard palate graft. The procedure was performed under local anesthesia (with infiltration of Lidocaine 2% + Epinephrine at 1:200,000) with sedation, or under general anesthesia. The patient was positioned in the supine position and radical excision was performed following the pre-operative markings. After accurate hemostasis, the harvesting of the Fricke flap was performed considering a 4:1 length-to-width ratio, customizing the flap according to size and shape of the defect. The donor site was located immediately above the eyebrow, with the maximal medial extent delineated by the supraorbital neurovascular bundle. The length of the flap was usually overestimated, later shortening the flap after transposition. The Fricke flap was then dissected from the underlying frontalis and orbicularis oculi muscles. Hemostasis was performed and the donor area was closed layer by layer with Monocryl 3.0 and Nylon 4.0. Finally, the hard palate graft was harvested at the paramedian level, preferably behind the incisive papilla to avoid bleeding due to anterior palatine peduncle damage [10] (Figure 1). The size of the graft was also slightly overestimated, anticipating a certain degree of contraction and retraction over time.

The sampling zone was infiltrated with local anesthesia containing Epinephrine to perform an accurate hydrodissection. If the surgery was performed under general anesthesia, a posterior pack was positioned in the oropharynx to prevent blood inhalation. The incision should reach the bone plane to avoid the graft being weak, and the detachment should be performed from all sides, taking care to control the bleeding that often occurs. Hemostasis was achieved through the compression of the donor area, as well as the selective coagulation of the borders. Coagulation on the bone was not performed because of the risk of necrosis and bucconasal fistulas. Hemostatic dressing was applied in all cases. After the placement of the packing, the harvested graft was placed on a damp sterile gauze, where it was customized and shaped specifically for the needs of the defect. Once the shaping was complete, the hard palate graft was placed to reconstruct the posterior lamella of the lower eyelid, where it was fixed into position by resorbable sutures to the residual medial mucotarsal margin. Finally, the Fricke flap was transposed and fixed to complete the reconstruction of the anterior lamella by using 3/0 Monocryl and 4/0 Nylon sutures. Antibiotics were administered twice a day for 6 days and corticosteroids were administered in cases of significant edema.

## 3. Results

**Figure 2:**
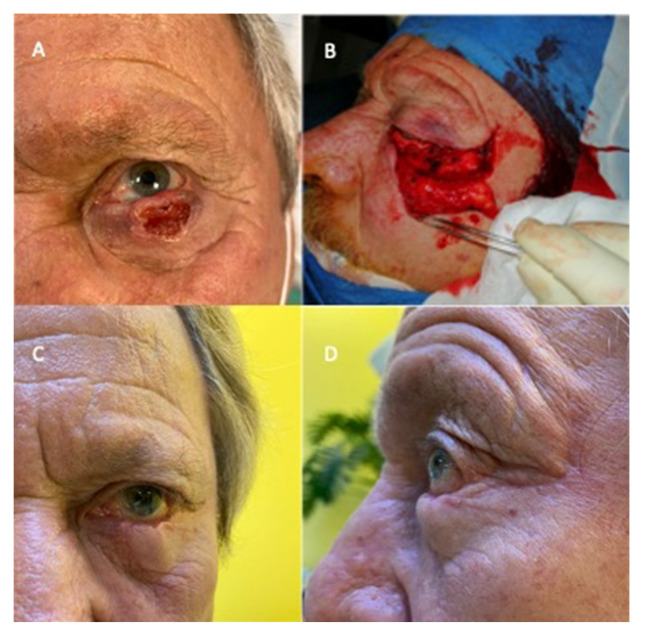
(**A**) Preoperative view, (**B**) size of defect, positioning of hard palate graft and flap harvesting, (**C**) frontal post-operative view, (**D**) lateral post-operative view.

**Figure 3:**
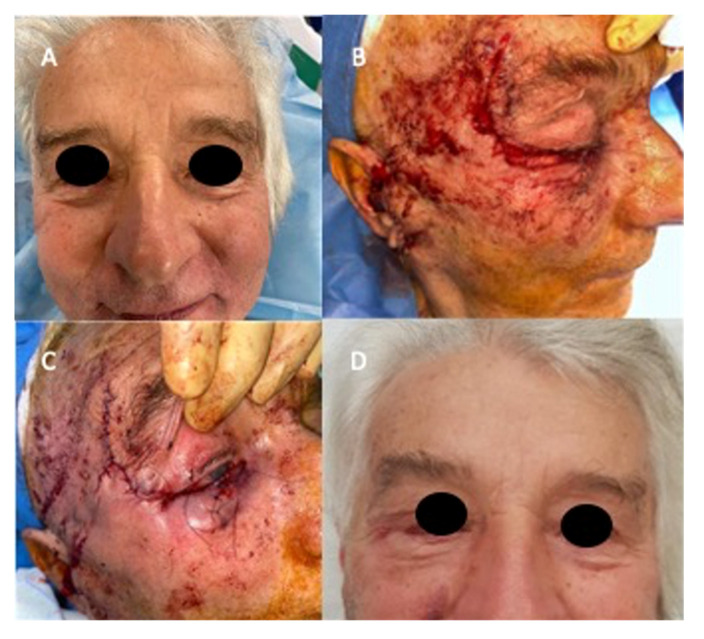
(**A**) Preoperative view, (**B**) incision and flap harvesting, (**C**) immediate post-operative view, (**D**) frontal post-operative view after suture removal.

**Figure 4:**
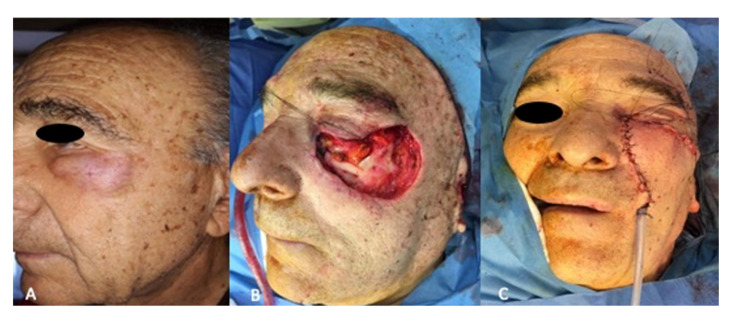
(**A**) Preoperative view, (**B**): intraoperative view after tumor resection, (**C**) immediate post-operative view.

A total of seven lower lid reconstructions with a Fricke flap and hard palate graft were performed; an immediate reconstruction was carried out in all the patients. (Figure 2, Figure 3 and Figure 4).

The clinical data, type of tumor, and excision margins are reported in Table 1.

Our sample population featured two women and five men. The mean age was 75.6 (range from 55 to 82). The mean dimension of the tumors was 1.7 × 1.7 cm. All featured histological types included non-melanoma skin cancers, and namely one Merkel cell carcinoma (MCC), two basal cell carcinomas (BCCs), and four squamous cell carcinomas (SCCs). Among the SCCs, one case was graded as G1, another as G2, and two as G3. Three non-melanoma skin cancers were located on the right side and four on the left side. Excisional margins were decided according to previously mentioned guidelines. In all cases, the tarsal palate was involved, requiring excision of the posterior lamella. In no case was the lacrimal point affected. No immediate complications were observed during the first post-operative month. Negative surgical margins were achieved in all cases. In the MCC case, laterocervical sentinel node biopsy was performed; the sentinel node was negative. All patients underwent dermatological and oncological follow up. During the late follow-up, lower lid ectropion was observed in one patient due to the development of a retracting scar. This complication was then managed with a revision surgery. No local cancer recurrence or nodal metastasis were detected until 2 years follow-up. The esthetic results were deemed satisfactory, and a value of 7/10 was assigned by five patients and of 6/10 by two patients on the VAS recorded at 1 year follow-up.

## 4. Discussion

Lower eyelid reconstruction is a delicate and difficult surgery, constituting a challenge for plastic surgeons due to the significant functional and esthetic implications of the eyelid. The best treatment should aim to achieve a radical excision with acceptable free margins and a satisfactory reconstruction, avoiding the development of ectropion and conjunctival chemosis. NSCC of the eyelid, especially SCC and MCC, is characterized by a high risk of relapse, lymphatic metastasis, and invasion of underlying structures. So, a radical excision is necessary. Given the constraints of the anatomy and function of the eyelids, excision with negative margins and reconstruction can be challenging. In cases of significant tissue invasion or metastasis, complete tumor removal may not be possible.

The standard treatment for all eyelid carcinomas is surgical excision with negative margins, although controversy still exists regarding the recommended margins for each specific malignancy [2]. Mohs micrographic surgery is a useful method of managing both BCC and SCC, when feasible. The benefits of this procedure is related to its capacity to determine margin control during excision and preserve the greatest amount of normal tissue. This is especially significant in the eyelid and medial canthus, where large excisions can have devastating effects on the appearance and function of the eyelids. Both frozen and permanent sectioning have been used for the histologic assessment of surgical margins. There does not seem to be a consensus in the literature regarding the preferred margin control technique for non-melanoma malignancies. In our practice, we prefer to perform permanent histological examination without using Mohs surgery due to the longer surgical time requested and costs.

Typical surgical margins for BCC are 2–3 mm, while more aggressive carcinomas such as Seb Ca, Merkel cell carcinoma, and melanoma require larger margins [13,14].

Surgical margins for more aggressive eyelid malignancies are typically larger than in cases of BCC. In a study by Peters et al. [13], the local control of Merkel cell carcinoma was achieved by wide excision with 5 mm margins. All eyelid lesions in this study were smaller than 2 cm and were confined to the eyelid. The authors concluded that histologic confirmation of tumor-free margins either through frozen section or Mohs micrographic surgery is a reasonable means of management [13]. Although there exists no consensus, a surgical margin of 5 mm when feasible is a reasonable goal for Seb Ca, Merkel cell carcinoma, and melanoma, which present both functional and esthetic challenges during larger reconstructions. Many practitioners recommend surgical excision with wider margins (up to 2 cm) in cases of Merkel cell carcinoma.

Several methods have been introduced considering size of the defect, age of the patient, and location of the lesions to provide optimal anatomical, esthetic, and functional results for the reconstruction of eyelids [4,15].

When a total lid reconstruction is planned, three main layers must be restored: (1) the external or skin, (2) the medial, and (3) the internal or conjunctival. In the elderly, large defects, even up to 40% of the lid, might possibly be directly repaired due to involutional skin laxity and excessive skin. Larger defects require reconstruction of the eyelid that should be as similar as possible to the basic anatomy of the lower lid, consisting of anterior and posterior lamella, to achieve the highest functional and esthetic results. The combination of both flap and graft components was developed for creating both lamellae.

Different techniques are usually used for lower eyelid reconstruction, such as local and locoregional flaps, free flaps, and skin grafts [16,17]. Local flaps are considered the gold standard of reconstruction of the lower lid [10]. In the recent years, several procedures have been developed, with acceptable functional and cosmetic outcomes and minimal complications. Considering anthropometric criteria in orbital and periorbital architecture can help the surgeon to achieve an acceptable cosmetic outcome and avoid any distortion in the appearance of the eyelids [16].

For anterior lamella reconstruction, the most widely used techniques include the Mustardé rotational flap, the Hughes transposition flap, the McGregor flap, and the Fricke flap. For posterior lamellar defects, the most used procedures are tarsal-conjunctival grafts, auricular or nasal (alar or septal) cartilage grafts, mucosal grafts, and fascia lata and Achilles tendon grafts [5]. The selected grafts need to be strong enough to withstand the forces that act on the lower eyelid and must provide a nonkeratinized posterior surface which can lead to corneal abrasion. The present article is intended to report our experience using a reconstructive technique constituting a combination of a hard palate graft, for the posterior lamella, and a Fricke flap, for the anterior one. The Fricke flap is a temporally based monopedicle forehead transposition flap which can be used in the reconstruction of lower/upper lids and lateral canthal defects [10] when primary closure is not possible. In the literature, many variants have been described, such as the modified Fricke’s cheek flap, where the flap is taken from the cheek instead then from the temporal region [11,12]. Some authors believe that using the cheek to harvest the flap has the advantage of providing a greater amount of tissue, without causing eyebrow elevation [9,10]. According to our experience, the Fricke flap shows better performance when planned from the temporal and supraorbital region instead of the cheek, due to the lower risk of ectropion [18,19], resulting from the traction that pushes the flap upward, rather than downwards. Using this flap abides by the principle of “replacing like with like” by using contiguous cosmetic subunits guaranteeing the same color, texture, and thickness of the skin. Among other advantages, this technique also allows for lateral cantal release when needed. The use of a myocutaneous upper lid flap is not recommended for the reconstruction of full-thickness defects due to its insufficient thickness to cover the graft. Although the Mustardé flap is often considered as the first option for lower lid reconstruction because of its better esthetic result, in elderly patients, the Fricke flap should be considered the first option because of the minor dissection required and the shorter operating times. A frequent late complication of the Mustardè flap is ectropion, especially in patients with poor skin laxity due to gravity and wound contraction. We usually resort to selecting the Mustardé flap for defects involving the lateral third of the lower lid in continuity with large cheek defects. Also, in cases in which patients cannot undergo general anesthesia harvesting, the Fricke flap is more feasible due to the minor dissection required. The excision of malignant tumors is typically performed under general anesthesia for extensive excisions, to ensure complete pain control, patient immobility, and optimal surgical conditions. However, in our experience, local anesthesia with sedation is considered depending on factors such as tumor size, patient comorbidities (some patients may not tolerate general anesthesia well), patient preference, and anxiety levels. So, in some cases, and also in relation to the anesthesiologist’s evaluation, we have to perform surgery under local anesthesia and sedation.

Concerning the posterior layer of lower eyelid reconstruction, many autogenous graft materials for lower lid support have been used, such as the fascia lata [20,21,22,23], nasal septal cartilage [24,25], upper lid tarsus [26,27], temporalis fascia [28], auricular cartilage [29,30], or palatal mucosa [6,31].

In the suspension procedure, using sling type materials such as the fascia lata or temporalis fascia, natural contact between the lower lid and the eyeball is difficult to achieve because tension in the lower lid presses the eyeball unevenly along the line of the sling. Although the upper lid tarsus is the ideal material for lower lid support, harvesting it necessitates surgery on the upper lid. A nasal septal cartilage graft is too stiff to secure the appropriate contact between the lower lid and the eyeball.

The hard palate graft is widely accepted as one of the best options due to the faster healing time of both the recipient and donor sites. It can be achieved after the administration of local anesthesia and the donor site heals in about 2 weeks with minimal care [9]. It is similar to the tarsus in terms of structural rigidity and it has a mucosal surface with a nonkeratinized epithelium comparable to the conjunctiva. Furthermore, it is possible to obtain large-sized grafts which are sufficient for correcting the entire length of the eyelid [6]. These characteristics provide good structural support to the reconstructed lid [3]. Hard palate graft is preferred in lower lid reconstructions and is not recommended for upper eyelids due to their major movement, which can lead to corneal abrasions caused by friction force. The Hughes tarso-conjunctival flap is another alternative method for the reconstruction of lower eyelid defects. However, this method must inevitably raid the unaffected upper eyelid for donor tissue, which is a major disadvantage. Also, in elderly patients, upper lid tarso-conjunctival flaps such as the Hughes procedure are not recommended because of the need for a two-stage procedure requiring a tarsorrhaphy with a 3–4-week waiting period in between, before achieving the final reconstruction. This is especially cumbersome in monocular patients, in whom occlusion of the visual axis with other techniques (such as Hughes flap) would be unacceptable [10]. Tarso-marginal grafts from the contralateral lid, as described by Hubner in 1976, are not widely used due to the high risk of severe complication such as epiphora, lagophthalmos, and lid notching [32]. Another technique described for the reconstruction of full-thickness defects of the lower lid is the island nasal chondromucosal flap [33]; this technique has the advantages of providing a one-stage reconstruction, but it is not recommended for larger defects involving the entire length of the lid. Furthermore, it is not recommended in elderly patients due to its difficult dissection using a loop and its long operating time; this technique needs a skin graft for anterior lamella reconstruction, with the risk of late-onset ectropion of the eyelid because of skin graft contracture.

The rotational advancement of a tarso-conjunctival cheek flap can wrap large defects as big as three-fourths of eyelid by providing a more physiologic structure and little disturbance to the donor site [34]. Functional and cosmetic problems due to deformity in the lateral canthus are drawbacks of this technique.

The sandwich technique comprises a muscle flap that is packed with free grafts in both sides [35]. Partial skin graft necrosis, ectropion, granuloma formation, lower lid retraction, notching, and adhesion in lateral canthus are reported complications.

The advantages of the proposed technique include the following: the color of the flap is similar to that of the lower lid, it presents a good consistency for supporting the eyelid, and it has an antigravity direction force which guarantees an excellent functional reconstruction; the flaps have a good and reliable blood supply and adequate and sufficient tissues. Tarsus reconstruction uses a hard palate graft to keep the eye open and prevents the development of ectropion. The flap donor area is closed primarily, or by graft. This technique provides a one-stage operation, which is a more acceptable surgical procedure for patients.

## 5. Conclusions

In conclusion, different valid surgical options are available for lower lid reconstruction; during preoperative planning, the surgeon has to consider not only the esthetic and functional outcomes but also the compliance of the patients, considering their comorbidity and surgical operative time. According to our experience, the combination of a Fricke flap and a hard palate graft is a good option for total lower lid reconstruction, giving the opportunity to obtain good results in a single-stage procedure, with a lower operative time and risk of bleeding; for this reason, it should be considered the best option in elderly and frail patients. This retrospective study presents several limitations, such as a small sample size, but this is mitigated by a standardized technique, a large number of variables, a close follow-up, and the absence of a control group. Comparative studies are necessary to confirm the superiority of this technique in elderly and frail patients.

## Figures and Tables

**Table 1:** Clinical data, histological type of tumors excised, and margins of excision.

Patient	Gender	Age	Histological Type	Lesion Diameter (l × h; cm)	Excision Margin (cm)	Free Margins	Side
1	F	77	BCC	2 × 1.8	0.5	0.3	Right
2	M	78	SCC (G2)	2 × 1.6	1	0.7	Right
3	M	80	BCC	1.8 × 1.8	0.5	0.3	Left
4	M	55	MCC	1 × 1	2	1.5	Left
5	M	82	SCC (G3)	1.5 × 2	1	0.8	Left
6	M	78	SCC (G3)	2 × 2	1	0.8	Left
7	F	79	SCC (G1)	1.5 × 1.5	0.6	0.6	Right
MEAN		75.6		1.7 × 1.7			

## Data Availability

The original contributions presented in this study are included in the article. Further inquiries can be directed to the corresponding author.
